# Continuous vs interrupted direct oral anticoagulants for minimal bleeding-risk surgical procedures—a systematic review and meta-analysis

**DOI:** 10.1016/j.rpth.2026.103419

**Published:** 2026-03-27

**Authors:** Alexander Xiang, Shakil Popatia, Sarah Lopes Sadafi, Megha Rao, Asim Shaikh, Mehrad Nowrouzi, Allen Li, Ali Eshaghpour, Mark Crowther

**Affiliations:** 1 Department of Medicine, McMaster University, Hamilton, Ontario, Canada; 2 Faculty of Medicine, University of Toronto, Toronto, Ontario, Canada; 3 Faculty of Health Sciences, McMaster University, Hamilton, Ontario, Canada; 4 Faculty of Medicine, University of Ottawa, Ottawa, Ontario, Canada; 5 Division of Vascular Surgery, Department of Surgery, University of Toronto, Toronto, Ontario, Canada

**Keywords:** factor Xa inhibitors, hemorrhage, perioperativecare, surgery, thrombosis

## Abstract

**Background:**

Evidence supporting the perioperative interruption of direct oral anticoagulants (DOACs) is varied, especially for procedures with minimal bleeding risk.

**Objectives:**

This review compared the risk of bleeding events and thromboembolism in patients undergoing minimal bleeding-risk procedures, in whom DOACs were interrupted or continued.

**Methods:**

Prospective, retrospective, and randomized trials examining a continuous or interrupted DOAC strategy in patients undergoing minimal bleeding-risk surgeries were included. Primary outcomes included the rates of venous or arterial thromboembolism and rates of major and minor bleeding events. Secondary outcomes included rates of surgical complications, mortality, and hospital length of stay.

**Results:**

We retrieved 24 studies (*N* = 8663 patients). Cardiac ablations were the most common procedure (*n* = 13), followed by pacemaker insertion (*n* = 4) and dental procedures (*n* = 4). Nineteen studies examined thromboembolic events, with a pooled odds ratio (OR) favoring continuous DOAC use (OR, 0.54; 95% CI, 0.33-0.91; *I*^2^ = 60%), although these results failed to persist in the high-quality randomized clinical trial data. Twenty studies examined bleeding events, with a significant reduction in major bleeds favoring continuous DOACs (OR, 0.57; 95% CI, 0.37-0.87; *I*^2^ = 0%), which failed to reach significance in the randomized data. There were no difference in minor bleeding (OR, 0.93; 95% CI, 0.69-1.24; *I*^2^ = 0%) events. No differences were noted in the rates of total complications or mortality.

**Conclusions:**

While our meta-analysis found an improvement in thrombotic and major bleeding events with continuous perioperative DOAC use, these results were likely subject to selection bias with no significant differences in all outcomes in the high-quality randomized trials. The findings in the randomized data noted no significant differences between a continuous and interrupted DOAC strategy, although further research is required to provide a more definitive conclusion.

## Essentials

• The risks of continuing oral anticoagulation for minimal bleeding risk surgeries are unknown.
• This study reviewed articles comparing continuing vs stopping anticoagulation in minimal risk surgery.
• High-quality data found no differences in bleeding and clotting between the 2 regimens.
• We found no difference between the 2 regimens in minimal risk surgery, but more data are needed.

## 1 Introduction

Direct oral anticoagulants (DOACs) are factor (F)Xa or direct thrombin inhibitors that have become the most common form of anticoagulation in the general population [1]. By 2019, ∼ 3.5 million individuals in the United States were receiving DOACs for venous thromboembolism (VTE) and nonvalvular atrial fibrillation [2]. DOAC treatment has been associated with fewer drug interactions, reduced monitoring requirements, and lower bleeding risks compared with vitamin K antagonists [3,4].

The high incidence of DOAC use presents a challenge in the periprocedural period, with over 250 000 patients each year assessed for preprocedural anticoagulant management prior to elective surgery [5]. The continued use of a DOAC in the perioperative period has been theorized to increase the risk for major and minor bleeding, reoperation, and postoperative complications such as hematoma formation that may increase morbidity and mortality [2,6]. Nevertheless, these adverse outcomes must be balanced against the risks of thromboembolism during anticoagulation interruption [7].

Considering the opposing risks associated with DOAC interruption in the perioperative period, current guidelines largely recommend interruption of DOAC therapy for most elective major surgical procedures [8,9]. Guidelines from sources such as Thrombosis Canada recommend stopping dabigatran, apixaban, and rivaroxaban for 1 day prior to a low- to moderate-risk procedure and 2 days for procedures with a high bleeding risk [8]. Longer discontinuations between 3 and 5 days for high bleeding–risk procedures have also been recommended, although decisions may also be influenced by other patient-specific factors, such as active cancer or a bleeding history [8,10,11].

While these recommendations regarding perioperative DOAC management have been widely adopted into clinical practice, the evidence supporting these decisions is limited, especially for procedures with a minimal risk of bleeding such as ablations, pacemaker insertions, and cataract surgery [12,13]. Most guidelines suggest continuing DOACs is safe, although withholding them on the day of the procedure is also acceptable. Given the limitations of the current literature on perioperative DOAC use in minimal risk of bleeding procedures, this systematic review and meta-analysis investigated the safety and effectiveness of a continuous DOAC regimen compared with an interrupted DOAC regimen for minimal-risk surgical procedures.

## 2 Methods

This systematic review and meta-analysis was reported according to the 2020 Preferred Reporting Instructions for Systematic Reviews and Meta-analysis (PRISMA) guidelines and was registered prospectively on the PROSPERO international database (CRD42024621729).

### 2.1 Data sources

We searched Ovid Medline, CENTRAL, EMBASE, CIHNAL, and trial registries (eg, clinicaltrials.gov) from inception until September 11, 2025, using a librarian-approved searched strategy, which used a combination of MeSH terms and keywords including but not limited to DOACs, novel oral anticoagulants, anticoagulants, FXa inhibitors, catheter ablations, and artificial pacemakers. A set sample of known included studies were used to validate the search strategy, the list of which is included in the Supplementary Appendix, eAppendix 3. The reference lists of eligible trials and relevant reviews were also be screened by authors for any additional studies. The full librarian-approved search strategy is available in the Supplementary Appendix, eAppendix 1. The search strategy was updated from October 7, 2024, and no new studies were included—a list of new studies that were considered for inclusion, along with the reasons for their exclusion, are available in the Supplementary Appendix, eAppendix 2.

### 2.2 Eligibility criteria and study selection

We included prospective cohort, retrospective cohort, and randomized clinical trials (RCTs) that (1) examined the use of both continuous and interrupted DOAC use in the perioperative period; (2) enrolled patients undergoing any form of minimal-risk bleeding procedure, including cardiac device implantation, catheter ablation, coronary angiography with radial access, minor dermatological procedures, cataract procedures, or minor dental procedures; and (3) evaluated any outcomes related to venous or arterial thromboembolism, rates of major bleeding, surgical complications, and mortality regardless of follow-up duration.

Bleeding risk was determined a priori from recently published literature, including the American College of Chest Physicians Clinical Practice Guideline on the Perioperative Management of Antithrombotic Therapy and the 2019 Subcommittee on Perioperative and Critical Care Thrombosis and Haemostasis of the International Society on Thrombosis and Haemostasis (ISTH) [2,8,14]. All studies evaluating these procedures, regardless of whether procedural bleed risk was reported, were included in the review. Minimal bleeding risk procedures included cardiac device implantation, coronary angiography using radial artery access, minor dermatologic procedures, cataract procedures, and minor dental procedures.

Minor dermatological procedures were classified as excision of basal and squamous cell skin cancers, actinic keratoses, and premalignant or cancerous skin nevi [8]. Minor dental procedures were classified as dental extractions, restorations, prosthetics, endodontics, dental cleanings, and fillings [8]. Articles examining any other surgical procedures were excluded. Non-English articles and conference abstracts or letters were excluded, and authors were contacted for clarifying eligibility or additional information when necessary.

An online platform, Covidence software (Covidence), was used for the literature screening and data extraction. Paired reviewers (A.J.X., S.P., S.L.S., M.R., A.S., and M.R.) screened titles or abstracts of identified citations and full texts of all potentially eligible studies independently and in duplicate. Studies were first evaluated based on their title and abstract, with the included studies undergoing full-text screening before being extracted for analysis. Any discrepancies were resolved by discussion or with third-party adjudication (A.L., A.E., A.J.X., and M.C.) if necessary.

### 2.3 Data extraction

Using standardized, pilot-tested forms on Excel (Microsoft), paired reviewers extracted each eligible trial independently and in duplicate, with discrepancies resolved with discussion and third-party resolution. Reviewers extracted study characteristics (eg, study design, setting, and funding source), patient characteristics (eg, age, sex, weight or body mass index, and American Society of Anesthesiologists status), type of procedure, DOAC used, period of interruption, and all patient-important outcomes—i.e. rates of thromboembolism, mortality, and major bleeding events.

DOAC interruption was classified into either continuous or interrupted. A continuous regimen continued the DOAC on the morning of the procedure, while an interrupted strategy discontinued 1 or more doses of anticoagulation. These durations of interruption were further classified based on the interruption interval—day of, 24 hours, 48 hours, or longer. Studies interrupting DOACs the day of provided a dose of anticoagulation the day prior to the procedure and the day after.

The primary efficacy outcome was a composite pooled rate of arterial and venous thromboembolism as defined by the study. The primary safety outcome was rate of bleeding events, both major and minor as defined by the study, irrespective of how it was defined. Secondary outcomes included hospital length of stay, mortality, and other surgical complications.

### 2.4 Statistical analysis

Continuous measures were converted to a common scale or unit on a domain-by-domain basis. If SDs for continuous outcomes were not reported directly, authors estimated SDs from SE, CIs, *P* values, IQR, or range. Continuous outcomes were pooled as weighted mean differences and all binary outcomes as odds ratio (OR) and risk difference, along with the associated 95% CIs.

All outcomes of interest that were reported by >1 study were pooled using a DerSimonian–Laird random-effects model. Heterogeneity was calculated using Cochrane chi-squared test and *I*^2^ statistic, which quantifies variation in study results explained by statistical heterogeneity. We considered 0% to 30% as low heterogeneity, 30% to 60% as moderate, 60% to 90% as substantial, and 90% to 100% as critical.

The authors planned 2 subgroup analyses a priori. The first subgroup analysis examined differences in reported outcomes stratified based on the type of minimal risk procedure. The second subgroup analysis examined outcome differences based on timeline of DOAC interruption (eg, day of and 1 day prior). Given the heterogeneity in study design between randomized and nonrandomized trials, the decision was made following the full-text extraction to complete a third subgroup analysis examining study outcomes based on type of study design, with separate analyses completed for RCTs, prospective, and retrospective cohort studies.

The credibility of significant subgroup effects were determined using the Instrument for assessing the Credibility of Effect Modification Analyses (ICEMAN) criteria [15]. If there were at least 10 studies available for meta-analysis, small-study effects were assessed by visual assessment for funnel plot asymmetry, Egger test for continuous outcomes, and Harbord’s test for binary outcomes. All statistical analyses were performed using Stata statistical software version 18 (StataCorp). All comparisons were 2-tailed using a threshold of *P* ≤ .05.

### 2.5 Study and evidence quality

For the randomized trials included in the study, the Cochrane Risk of Bias 2.0 tool was used to assess the quality of evidence [16]. For nonrandomized studies, the Risk of Bias In Non-Randomized Studies of Interventions Version 2 (ROBINS-I v2) tool was used to grade the quality of evidence [17,18]. The authors (A.J.X., S.P., S.L.S., M.R., A.S., and M.R.) completed all risk of bias assessments in duplicate, with any discrepancies resolved using third party adjudication. Overall certainty of evidence was assessed using the Grading of Recommendations, Assessment, Development, and Evaluations (GRADE) tool [19].

The body of evidence for each outcome measure was assessed to be of high, moderate, low, or very low certainty. The degree of certainty indicates confidence that the true effect is close to the estimated effect. High certainty typically results from well-executed RCTs, moderate from RCTs with limitations or inconsistencies, low from nonrandomized data, and very low for randomized or nonrandomized data with methodological issues or imprecision [19].

## 3 Results

**Figure 1:**
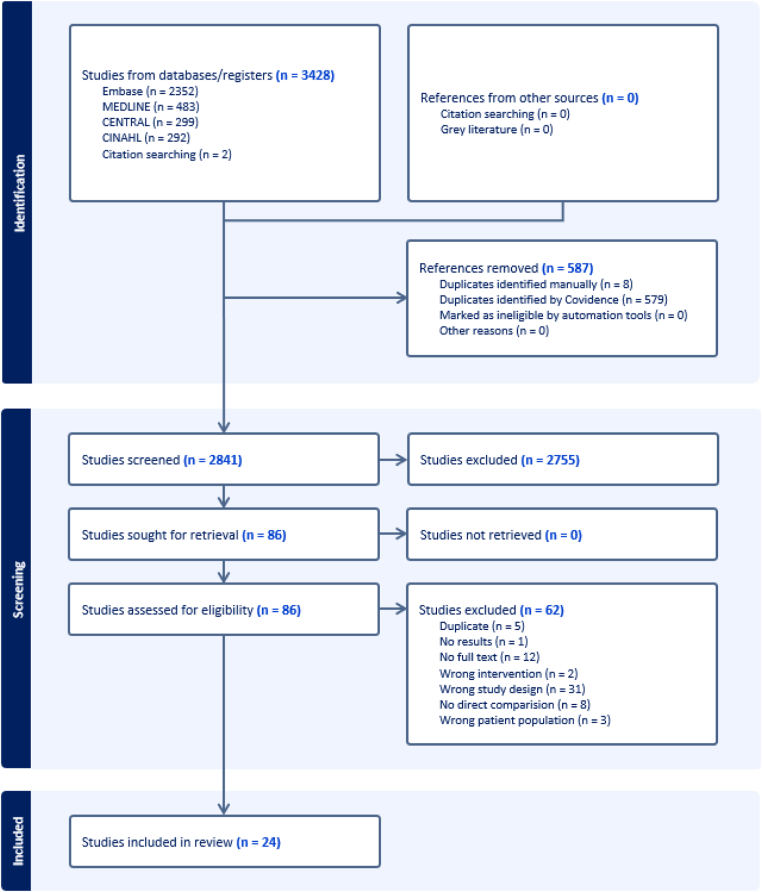
PRISMA flowchart of included studies.

Our search strategy yielded a total of 3428 total results, with Covidence removing 587 duplicate studies. Title and abstract screening excluded 2755 studies, with 86 full texts assessed for eligibility. Full-text screening removed 62 studies, leaving 24 studies included in the final review (*N* = 8663 patients). A flowchart depicting the process of study selection is illustrated in Figure 1.

### 3.1 Study characteristics

Of the studies included in the final analysis, 8 were retrospective cohort studies (*n* = 2624 patients) [[20], [21], [22], [23], [24], [25], [26], [27]], 8 were prospective cohort studies (*n* = 2928 patients) [[28], [29], [30], [31], [32], [33], [34], [35]], and 8 were randomized controlled trials (*n* = 3111 patients) [12,[36], [37], [38], [39], [40], [41], [42]]. Moreover, 11 (*n* = 3541) of the 24 studies were interventional, while the remaining 13 (*n* = 3460) were observational. Among these observational trials, only 2 studies reported an a priori periprocedural DOAC management protocol, with both studies leaving the decision to the discretion of the attending physician [30,34]. The most common countries of origin for the included studies were Japan (*n* = 8 studies), the United States (*n* = 4), Germany (*n* = 2), South Korea (*n* = 2), and Italy (*n* = 2). The most common minimal bleeding-risk procedures were ablations (*n* = 13 studies), followed by pacemaker insertion (*n* = 4) and dental procedures (*n* = 4). The most common DOACs in each study included apixaban (*n* = 2991 patients), rivaroxaban (*n* = 2479 patients), dabigatran (*n* = 2042 patients), and edoxaban (*n* = 786 patients). Most patients were on a DOAC for atrial fibrillation (*n* = 8448 patients).

Each study also slightly varied in the timing of their interrupted DOAC regimen—6 studies interrupted the DOAC dose the morning of the procedure, 9 studies interrupted the DOAC dose 24 hours prior, 5 studies interrupted the DOAC dose at 48 hours, and 1 study interrupted the DOAC dose 72 hours prior to the procedure. The most commonly used definition of major bleeding was the ISTH criteria (*n* = 9 studies), with the remaining studies using study-specific definitions. All bleeding events not classified as major bleeding events were defined as minor bleeding events. A summary of the key baseline characteristics of all included studies is available in Table 1.

**Table 1:** Baseline characteristics of included studies.

Study, year	Country	Study design	Procedure type	Sample size	DOAC interruption	Bridging	Mean age (y)	Female (%)	DOAC used		
									Rivaroxaban	Apixaban	Other
Ando et al [36], 2019	Japan	RCT	Ablation	97	24 h	No	66.7	22.7	0	97	0
Aoyama et al. [28], 2019	Japan	Prospective	Ablation	272	24 h	Yes	65.8	36.76	77	101	94
Black-Maier et al. [20], 2017	USA	Retrospective	Pacemaker	60	NR	No	70.5	NR	NR	NR	NR
Birnie et al. [12], 2018	Canada	RCT	Pacemaker	662	48 h	No	73.8	27.6	212	246	203
Cheung et al. [21], 2019	Hong Kong	Retrospective	Cataract	66	48 h	NR	79	56.06	NR	NR	NR
Creta et al. [29], 2023	UK	Prospective	Pacemaker	1326	48 h	No	74.14	35.6	452	652	222
Gjermeni et al. [22], 2023	Germany	Retrospective	Ablation	1290	48 h	Yes	62.3	31.2	399	194	697
Izzetti et al. [30], 2024	Italy	Prospective	Dental	49	24 h	No	72.2	42.9	17	16	16
Kwak et al [23], 2019	South Korea	Retrospective	Dental	120	72 h	No	69.43	44.17	41	45	19
Lababidi et al. [24], 2018	Australia	Retrospective	Dental	43	NR	No	72	53.49	26	14	3
Miller and Miller [25], 2018	USA	Retrospective	Dental	12	48 h	No	70.6	16.7	7	2	3
Muller et al. [31], 2016	Germany	Prospective	Ablation	106	24 h	Yes	63.23	36	NR	NR	NR
Nagao et al. [37], 2019	Japan	RCT	Ablation	200	Day of	No	70	37	NR	98	102
Nakamura et al. [33], 2018	Japan	Prospective	Ablation	123	Day of	No	65	31.7	32	36	55
Nakamura et al. [32], 2019	Japan	RCT	Ablation	846	Day of	No	65	29.4	311	242	291
Nakamura et al. [38], 2019	Japan	Prospective	Ablation	333	Day of	No	64.3	33.3	122	48	163
Osawa et al. [26], 2022	Japan	Retrospective	Ablation	213	24 h	No	58	17.4	116	24	73
Petzl et al. [34], 2021	Austria	Prospective	Ablation	237	24 h	No	59.6	29.7	95	72	60
Reynolds et al. [39], 2018	USA	RCT	Ablation	295	Day of	No	63.5	32.9	0	295	0
Ricciardi et al. [40], 2018	Italy	RCT	Pacemaker	101	24 h	No	76	34.7	33	31	37
Sheikh et al. [27], 2021	USA	Retrospective	Mix	820	24 h	No	69.8	43.78	261	547	12
Van der Wall et al. [35], 2021	Netherlands	Prospective	Mix	479	NR	Mixed	66.51	34.56	0	0	479
Yamaji et al. [41], 2019	Japan	RCT	Ablation	584	Day of	No	65.7	27.4	174	117	293
Yu et al. [42], 2019	Korea	RCT	Ablation	326	24 h	Mixed	58.3	25.46	104	114	108

DOAC, direct oral anticoagulant; NR, not reported; RCT, randomized controlled trial.

### 3.2 Primary efficacy outcomes

**Figure 2:**
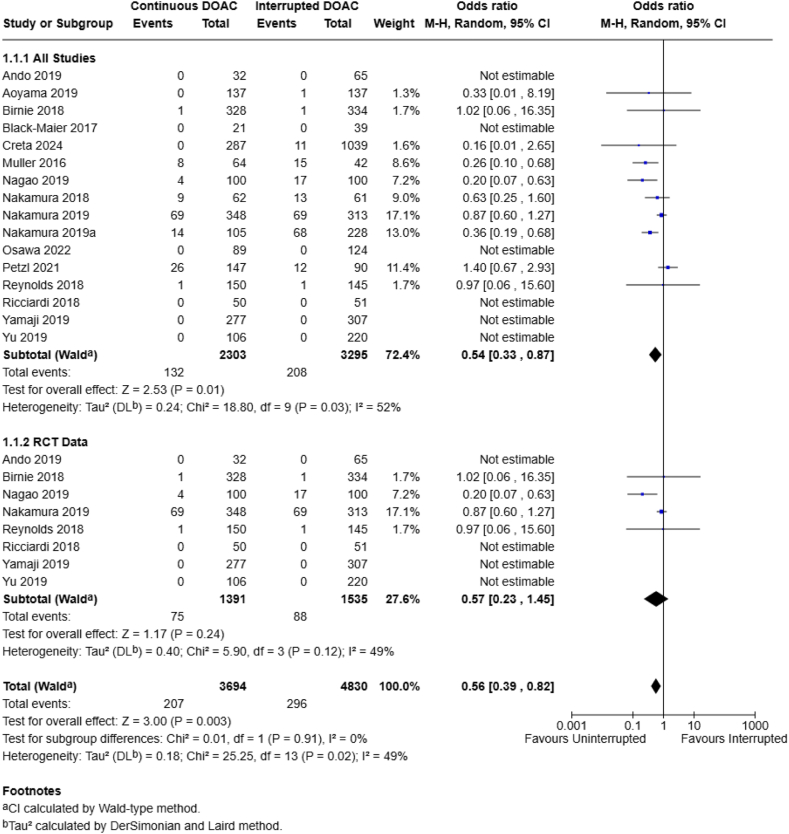
Continuous vs interrupted direct oral anticoagulant (DOAC) use—thrombotic complications.

Thrombotic events were assessed by 19 studies (*n* = 8164 patients). When all end points were included in the final analysis, a statistically significant improvement in thrombotic events was noted favoring the continuous DOAC regimen (5.7% vs 6.3%; OR, 0.54; 95% CI, 0.33-0.87; *I*^2^ = 52%) (Figure 2). A similar effect direction was noted when isolating for thrombotic events at 30 days, although this did not reach statistical significance (4.9% vs 4.5%; OR, 0.57; 95% CI, 0.28-1.16; *I*^2^ = 34%). Both end points were noted to have a moderate amount of heterogeneity.

**Figure 3:**
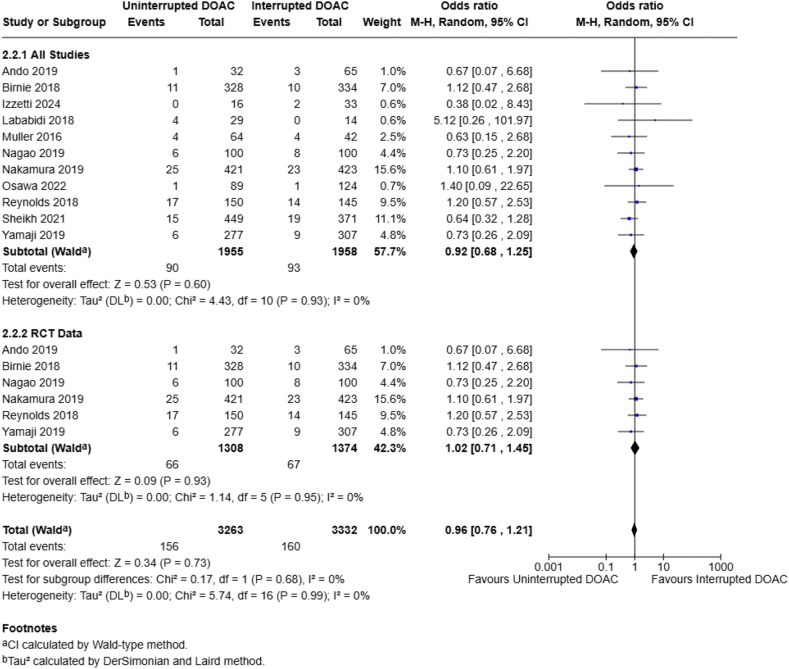
Continuous vs interrupted direct oral anticoagulant (DOAC) use—minor bleeding events.

**Figure 4:**
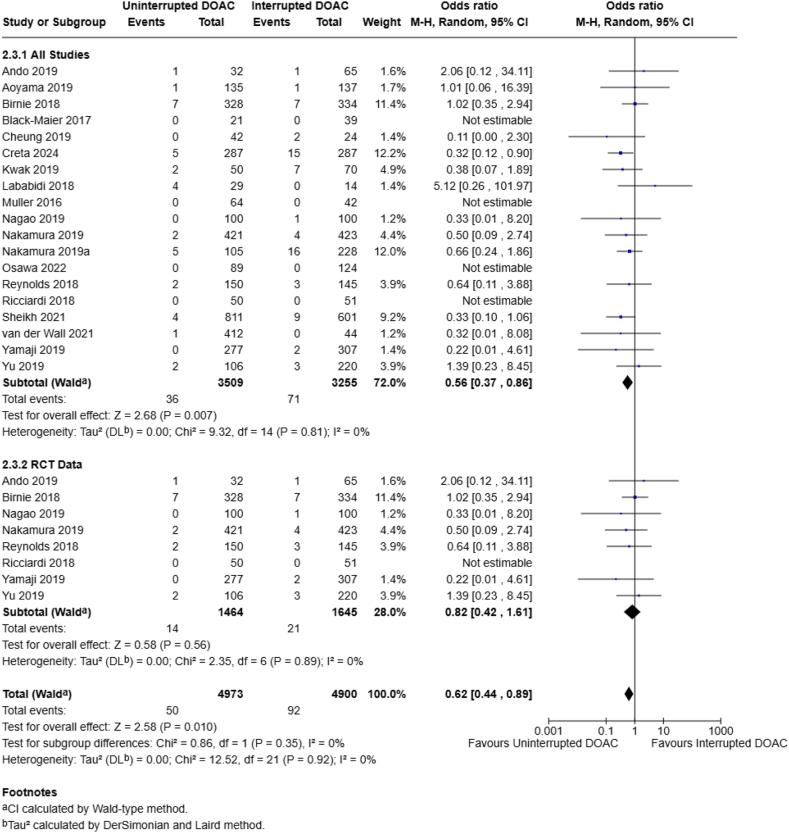
Continuous vs interrupted direct oral anticoagulant (DOAC) use—major bleeding events.

Bleeding events were assessed by 20 studies (n = 7001 patients). When all end points and bleeding events were included in the analysis, there was no statistically significant difference in the rates of overall bleeding events between a continuous and interrupted DOAC regimen (2.4% vs 3.2%; OR, 0.79; 95% CI, 0.62-1.01; *I*^2^ = 0%) (Figures 3 and 4). Nineteen studies examined major bleeding events (*n* = 6172 patients), with a statistically significant pooled OR favoring the continuous DOAC group (1.0% vs 2.2%; OR, 0.57; 95% CI, 0.37-0.87; *I*^2^ = 0%) (Figure 4). Lastly, 12 studies specifically examined minor bleeding events (*n* = 4185 patients), noting no statistically significant difference between the continuous or interrupted DOAC groups (4.6% vs 4.8%; OR, 0.93; 95% CI, 0.69-1.24; *I*^2^ = 0%) (Figure 3) at all end points. All pooled analyses in the bleeding events group did not have any observed heterogeneity.

### 3.3 Secondary analyses

**Figure 5:**
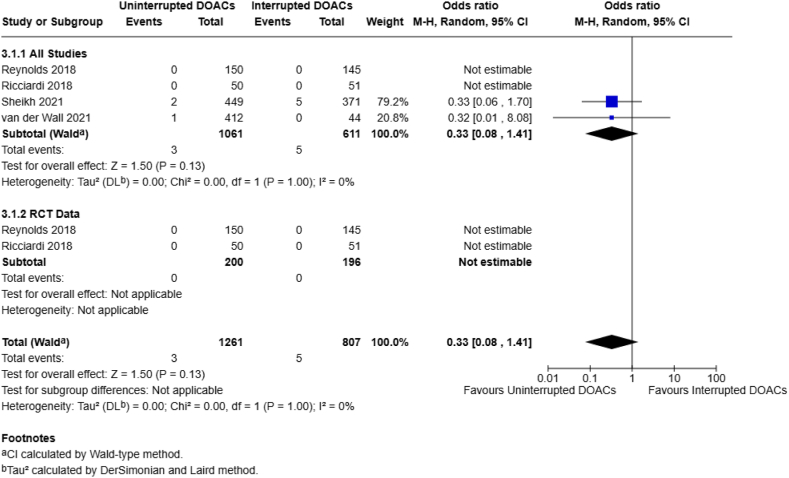
Continuous vs interrupted direct oral anticoagulant DOAC use—mortality.

**Figure 6:**
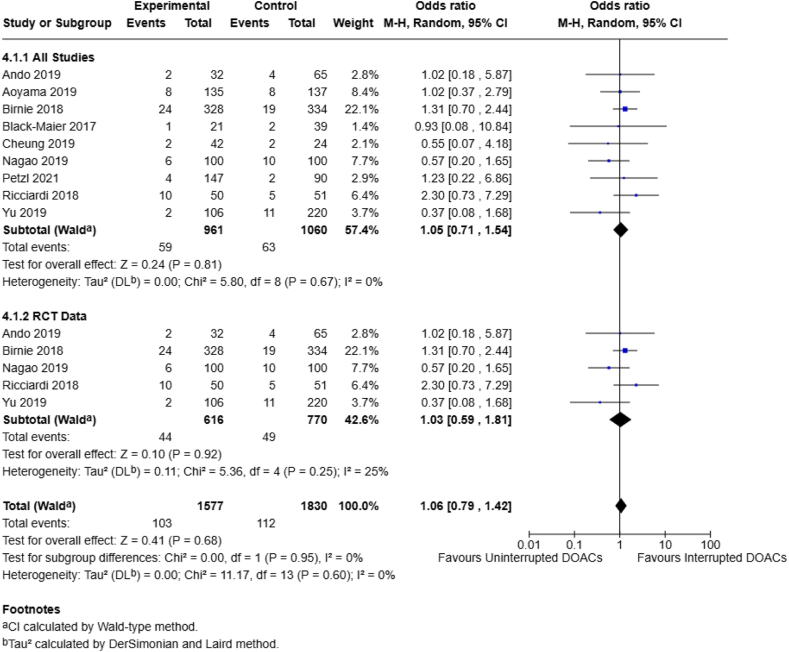
Continuous vs interrupted direct oral anticoagulant (DOAC) use—total complications.

Given the nature of minimal bleeding-risk surgeries, mortality was only assessed by 4 different studies (*n* = 1672 patients). A pooled analysis noted no significant difference between the continuous and interrupted DOAC groups (0.3% vs 0.8%; OR, 0.33; 95% CI, 0.08-1.41; *I*^2^ = 0%) (Figure 5), with no observed heterogeneity at all end points, albeit with a low event rate. Total complications were assessed by 9 studies (*n* = 2021 patients), with a pooled OR that failed to reach statistical significance, with no observed heterogeneity (6.1% vs 5.9%; OR, 1.05; 95% CI, 0.71-1.54; *I*^2^ = 0%) (Figure 6).

### 3.4 Subgroup analyses

Two separate subgroup analyses were organized a priori, with a third planned after the full-text screening stage. The first subgroup isolated studies based on study design. Across 8 randomized studies, there was a no differences in thrombotic (OR, 0.53; 95% CI. 0.17-1.64), major bleeding (OR, 0.82; 95% CI, 0.42-1.61), and minor bleeding (OR, 1.02; 95% CI, 0.71-1.45) events between the 2 anticoagulation strategies. Eight prospective nonrandomized studies noted a significant reduction in thrombotic events (OR, 0.50; 95% CI, 0.27-0.92), but no difference in major (OR, 0.65; 95% CI, 0.26-1.65) and minor (OR, 0.99; 95% CI, 0.62-1.60) bleeding events. Lastly, 8 retrospective nonrandomized studies revealed no differences in thrombotic (OR, 0.56; 95% CI, 0.23-1.38), major bleeding (OR, 0.43; 95% CI, 0.15-1.19), and minor bleeding (OR, 0.74; 95% CI, 0.38-1.45) events. There was a statistically significant difference in total bleeding events noted between RCT data (OR, 0.96; 95% CI, 0.70-1.32) and nonrandomized data (OR, 0.56; 95% CI, 0.38-0.81; *P* = .03). No differences were noted in all secondary outcomes.

The second subgroup analysis examined differences in reported outcomes stratified based on the type of minimal risk procedure. Studies examining ablations noted a significant reduction in thrombotic events (OR, 0.53; 95% CI, 0.34-0.84), while studies focusing on pacemaker insertions noted a similar, but not significant, effect direction (OR, 0.41; 95% CI, 0.06-2.95). Studies examining ablations noted no difference in major (OR, 0.70; 95% CI, 0.37-1.34) or minor (OR, 0.98; 95% CI, 0.69-1.39) bleeding events. There were no significant differences between major (OR, 0.57; 95% CI, 0.18-1.76) and minor (OR, 1.12; 95% CI, 0.47-2.68) bleeding events in the pacemaker population. Lastly, no significant differences in major (OR, 1.02; 95% CI, 0.08-12.70) and minor (OR, 1.44; 95% CI, 0.11-18.47) bleeding events in the dental procedure group were noted. No difference was noted in any secondary outcomes.

The third subgroup analysis isolated studies based on the duration of DOAC interruption. Studies that interrupted DOACs the morning of the procedure, in comparison with the continuous group, noted a significant decrease in thrombotic events (OR, 0.51; 95% CI, 0.29-0.93), favoring the continuous DOAC group. No significant differences in major (OR, 0.56; 95% CI, 0.27-1.19) and minor (OR, 1.01; 95% CI, 0.68-1.49) bleeding events were also noted. Studies that interrupted DOACs for 24 hours noted no differences in thrombotic events (OR, 0.68; 95% CI, 0.24-1.91) and major (OR, 0.66; 95% CI, 0.27-1.60) and minor (OR, 0.72; 95% CI, 0.43-1.20) bleeding events in comparison with a continuous regimen. Compared with studies interrupting DOACs for 48 hours, the continuous DOAC group noted a significant reduction in thrombotic events (OR, 0.41; 95% CI, 0.17-0.96), with no differences in major (OR, 0.48; 95% CI, 0.17-1.36) bleeding events. No difference was noted for all secondary outcomes.

Lastly, based on a reviewer feedback, a final a post hoc analysis investigating symptomatic strokes or transient ischemic attack was conducted, which was unfortunately limited due to a low number of events. A reduction in stroke or transient ischemic attack was observed favoring the continuous DOAC group, although this result was underpowered and failed to reach statistical significance (OR, 0.73; 95% CI, 0.26-2.04; *P* = .55; *I*^2^ = 0). A summary table with the results of all pooled analysis along with each of the GRADE analysis ratings is available in Table 2. A summary of the subgroup analyses, along with the ICEMAN criteria describing the credibility of each subgroup analysis is available in the Supplementary Appendix, eTables 2 and 3.

**Table 2:** Summary of analysis and GRADE ratings.

End point	Time point(s)	Intervention	Comparator	Studies (patients)	Effect (95% CI)	*P*	*I*^2^ (%)	GRADE certainty	Justification
Thrombotic events	All	Continuous DOAC regimen	Interrupted DOAC regimen	16 studies (*n* = 5598)	OR, 0.54 (0.33-0.87)	.01	52	Very low	Rated down: (1) 11 of 16 studies at high risk of bias; (2) moderate heterogeneity; (3) no indirectness; (4) no imprecision; (5) no publication bias (*P* = .25)
	30 Days	Continuous DOAC regimen	Interrupted DOAC regimen	9 studies (*n* = 3834)	OR, 0.57 (0.28-1.16)	.12	34	Very low	Rated down: (1) 7 of 9 studies at high risk of bias; (2) moderate heterogeneity; (3) no indirectness; (4) no imprecision; (5) no publication bias (*P* = .3)
All bleeding events	All	Continuous DOAC regimen	Interrupted DOAC regimen	20 studies (*n* = 7001)	OR, 0.79 (0.62-1.01)	.06	0	Low	Rated down: (1) 15 of 20 studies at high risk of bias; (2) no heterogeneity; (3) no indirectness; (4) no imprecision; (5) no publication bias (*P* = .58)
Major bleeding events	All	Continuous DOAC regimen	Interrupted DOAC regimen	19 studies (*n* = 6172)	OR, 0.57 (0.37-0.87)	.009	0	Low	Rated down: (1) 14 of 19 studies at high risk of bias; (2) no heterogeneity; (3) no indirectness; (4) no imprecision; (5) no publication bias (*P* = .53)
Minor bleeding events	All	Continuous DOAC regimen	Interrupted DOAC regimen	12 studies (*n* = 4185)	OR, 0.93 (0.69-1.24)	.61	0	Low	Rated down: (1) 10 of 12 studies at high risk of bias; (2) no heterogeneity; (3) no indirectness; (4) no imprecision; (5) no publication bias (*P* = .91)
Total complications	Discharge	Continuous DOAC regimen	Interrupted DOAC regimen	9 studies (*n* = 2021)	OR, 1.05 (0.71-1.54)	.81	0	Low	Rated down: (1) 6 of 9 studies at high risk of bias; (2) no heterogeneity; (3) no indirectness; (4) no imprecision; (5) no publication bias (*P* = .32)
Mortality	Discharge	Continuous DOAC regimen	Interrupted DOAC regimen	4 studies (*n* = 1672)	OR, 0.33 (0.08-1.41)	0.13	0	Low	Rated down: (1) 3 of 4 studies at high risk of bias; (2) no heterogeneity; (3) no indirectness; (4) no imprecision; (5) no publication bias (*P* = .45)

### 3.5 Risk of bias

Risk of bias in the 16 nonrandomized studies included in the final analysis was conducted using the ROBINS-I v2 tool. Of these studies, a total of 15 were judged to be at a high risk of bias [[20], [21], [22], [23], [24], [25], [26], [27], [28],[30], [31], [32], [33], [34], [35]]. The remaining 1 nonrandomized study was judged to be at a moderate risk of bias [29]. Within the nonrandomized studies, high risk of bias ratings was most often a result of bias due to confounding variables and a lack of adjustment for these factors in the statistical analysis. Risk of bias in the remaining 8 RCTs was completed using the Cochrane ROB 2.0 tool [43]. Of the included RCTs, 4 were noted to be at high risk of bias [[36], [37], [38], [39]]; 2 were noted to have some concerns [40,41]; and 2 were noted to be at low risk of bias [12,42]. The concerns in the high risk of bias studies were noted in the randomization process and the selection of reported results. The risk of bias judgments for all studies, both for each individual domains and overall, for each study are summarized in Tables 3 and 4, respectively.

**Table 3:** ROBINS-I v2 risk of bias assessment for nonrandomized studies.

Study, year	Domain 1	Domain 2	Domain 3	Domain 4	Domain 5	Domain 6	Domain 7	Overall
Aoyama et al. [28], 2019	−	−	+	+	−	+	+	X
Black-Maier et al. [20], 2017	X	−	−	+	−	+	−	X
Cheung et al. [21], 2019	X	−	+	−	−	+	−	X
Creta et al. [29], 2023	−	+	+	+	+	+	+	−
Gjermeni et al. [22], 2023	X	−	−	+	−	+	−	X
Izzetti et al. [30], 2024	−	−	−	+	+	−	−	X
Kwak et al [23], 2019	X	−	+	+	+	+	+	X
Lababidi et al. [24], 2018	X	+	−	−	−	−	−	X
Miller and Miller [25], 2018	−	−	+	−	+	+	+	X
Muller et al. [31], 2016	−	+	+	−	+	+	+	X
Nakamura et al. [32], 2019	X	+	−	−	−	+	+	X
Nakamura et al. [37], 2018	X	−	+	X	+	+	−	X
Osawa et al. [26], 2022	X	−	+	−	+	+	+	X
Petzl et al. [34], 2021	−	−	+	+	−	+	+	X
Sheikh et al. [27], 2021	X	−	+	+	+	+	−	X
Van der Wall et al. [35], 2021	X	+	+	−	−	−	+	X
Domain 1—Bias due to confounding Domain 2—Bias in classification of interventions Domain 3—Bias due to selection of participants Domain 4—Bias due to deviations from intended interventions Domain 5—Bias due to missing data Domain 6—Bias in measurement of outcome Domain 7—Bias in selection of the reported results						Judgment		
						X		Serious
						−		Moderate
						+		Low
						?		No information

**Table 4:** Cochrane ROB 2.0 assessment for randomized trials.

Study, year	Domain 1	Domain 2	Domain 3	Domain 4	Domain 5	Overall
Ando et al [36], 2019	X	−	+	+	−	X
Birnie et al. [12], 2018	+	+	+	+	+	+
Nagao et al. [37], 2019	−	+	+	+	−	X
Nakamura et al. [38], 2019	−	+	+	−	−	X
Reynolds et al. [39], 2018	X	+	+	−	X	X
Ricciardi et al. [40], 2018	+	+	+	+	−	−
Yamaji et al. [41], 2019	+	+	+	+	−	−
Yu et al. [42], 2019	+	+	+	+	+	+
Domain 1—Risk of bias arising from the randomization process Domain 2—Risk of bias due to deviations from the intended interventions Domain 3—Missing outcome data Domain 4—Risk of bias in measurement of the outcome Domain 5—Risk of bias in selection of the reported results				Judgment		
				X	High	
				-	Some concerns	
				+	Low	
				?	No information	

### 3.6 GRADE analysis

GRADE analysis of each outcome noted a very low certainty of evidence for thrombotic events and all bleeding events. Low-certainty evidence was noted for minor and major bleeding events, total complications, and mortality. All outcomes were rated down for both a large number of nonrandomized studies and for the presence of high risk of bias studies in the analysis. Thrombotic events and all bleeding events were further rated down due to moderate levels of heterogeneity in the pooled analysis. There was no evidence of imprecision, indirectness, or publication bias in each of the pooled outcomes. None of the outcomes in this review were rated upward for large effect sizes or dose-response gradients. Finally, there was insufficient evidence to suggest that the plausible confounding in the included trials would reduce the demonstrated effect or suggest a spurious effect when the results show no effect. A summary of each outcome, along with the associated GRADE results, are presented in Table 2.

## 4 Discussion

This systematic review and meta-analysis compared the rate of thrombotic and bleeding complications in patients on a continuous vs interrupted DOAC regimen undergoing minimal bleeding-risk procedures. While there was a significant reduction in the rate of composite thrombotic outcomes favoring the continuous DOAC regimen, this failed to reach significance when isolating for the high-quality RCT data and was primarily noted among the low-quality observational data. Low-certainty evidence found no differences between the 2 regimens in the rates of minor bleeding events, total surgical complications, and mortality. Finally, there was also a signal suggestive of reduced risk of major bleeding events in patients undergoing continuous anticoagulation across all studies. These effects failed to reach significance in the randomized data, which noted no differences in rates of thrombotic complications, major, and minor bleeding events. Finally, there were significant subgroup differences in total bleeding events between the randomized and nonrandomized data, further hinting at the impact of potential selection bias on key study outcomes.

It is important to take into consideration selection bias when interpreting the results of our review—in the nonrandomized data, patients at a low risk of bleeding were likely offered continuous anticoagulation regimes whereas high-risk bleeding patients had their anticoagulation held. The findings in the thrombotic event outcome may have been affected in a similar manner. In the observational data, the healthier patients at less risk of perioperative thrombotic complications may have been more likely to be offered a continuous DOAC regimen—this was acknowledged by one of the included studies by Sheikh et al. [27], who noted that more comorbid patients tended to be offered an interrupted DOAC regimen [27]. These concerns are further supported by the fact that the observed benefit was lost in the subanalysis of randomized studies, likely due to study blinding that prevented different physician-specific care or treatment allocation when the patient’s anticoagulation status was known.

All but 2 nonrandomized studies failed to report an a priori protocol, specifying how participants were selected into the continuous or interrupted regimen. As such, the subgroup examining the randomized controlled trials identified in our search appears to be the most robust data set, in contrast to the poor risk of bias judgments noted in the ROBINS-I v2 analysis. There are some additional limitations to consider. Two of the primary outcome measures—thrombotic events and overall bleeding events—were subject to moderate levels of heterogeneity. Plausible explanations for this include study-specific factors, such as type of procedure, blinding, timing of anticoagulation, follow-up duration, and outcome criteria, limiting the generalizability of pooled effects. The majority of included studies did not report any data related to race and ethnicity, which may have potentially impacted rates of venous thromboembolism [44]. Lastly, our study also did not separate patients based on their specific DOAC, which may have impacted the results given their diverse half-lives that could have influenced the results of this review.

Regardless, there are other key strengths of our review that must be taken into consideration. First, our review was able to capture 11 additional randomized and nonrandomized trials compared with previous reviews examining perioperative anticoagulation strategies, increasing the sample size and power of the pooled effect measures. Not only were overall pooled measures calculated across various anticoagulation strategies and procedure types, but separate subgroup analyses focusing on duration of interruption, procedure type, and study design were also conducted to evaluate the impact of other modifying variables. It is also key to note that even among the randomized data, the pooled effect measures failed to indicate a harmful effect of continuing perioperative DOAC use, in terms of both thromboembolic and bleeding outcomes. Lastly, a broad range of patient-important outcomes including arterial and venous thromboembolism, major and minor bleeds, hospital length of stay, and complication rates were pooled with a large patient sample, providing narrow CIs and precise estimates.

The results of this study both reinforce and add to existing knowledge from the current literature. A small-scale review conducted by Mendoza et al. [45] comparing a continuous and interrupted DOAC strategy for cardiac device implantation noted no difference in the rates of thromboembolism or clinically significant pocket hematoma. A second smaller scale review by Basu-Ray et al. [46] examining catheter ablations noted fewer silent cerebral events with a continuous strategy, with similar rates of major cerebrocardiovascular events and bleeding events between the 2 groups. Lastly, a large-scale review comparing perioperative DOAC and vitamin K antagonist strategies by Ha et al. [47] noted similar rates of major bleeding events between a continuous and interrupted DOAC regimen in atrial fibrillation ablations, although no comparison between thrombotic events were made [47]. Each of the described studies were similar to our review in that a continuous DOAC strategy was found to be noninferior to an interrupted strategy, with some studies further supporting a reduction in thrombotic events favoring the continuous DOAC group. Trends in perioperative anticoagulation is further extending to procedures such as transcatheter aortic valve implantation, with mixed evidence noting either minor benefits favoring a continuous regimen or no difference between the 2 groups [48].

Future research should focus on several avenues to both reinforce the current conclusions from this review and expand to new, clinically relevant dilemmas. First, more RCTs using a randomized, blinded methodology would is critical to ensure that knowledge of the patient’s anticoagulation status is not influencing outcome ascertainment. Arterial and venous thromboembolism should be separated into different outcomes, along with major and minor bleeding events. Further, more nuanced outcomes such as silent cerebral events should be evaluated by future trials, as these events are often found after minor procedures such as ablations and increase the risk of future cerebral thrombotic events [49].

## 5 Conclusions

While our overall analysis found very low–certainty evidence, suggesting a reduction in thrombotic and major bleeding events with continuous DOAC use in minimal bleeding-risk procedures, these effect measures were limited by study design and potential selection bias. Among the high-quality randomized data included in our review, there were no observed differences between a continuous and interrupted DOAC strategy in all outcome measures. These findings suggest that both a continued and interrupted DOAC strategy may be acceptable in the perioperative period for patients undergoing minimal bleeding-risk procedures, although higher-quality studies are needed to establish a definitive conclusion.
