# Supplementary material for: Continuous vs interrupted direct oral anticoagulants for minimal bleeding-risk surgical procedures—a systematic review and meta-analysis
Source: Res Pract Thromb Haemost. 2026 Mar 27;10(3):103419. doi: 10.1016/j.rpth.2026.103419 (PMC13099467; doi:10.1016/j.rpth.2026.103419)
Supplement: Supplementary Material [file mmc1.docx]

**Perioperative DOACs SR Supplement**

Contents

[Supplementary Appendix 1 Search Strategy 2](#_Toc227057942)

[Supplementary Table 1 – Summary Table of Subgroup Analyses 6](#_Toc227057943)

[Supplementary Table 2 – ICEMAN Table for Credibility of Subgroup Analyses 7](#_Toc227057944)

[Supplementary Table 3 – ICEMAN Table for Credibility of Subgroup Analyses 8](#_Toc227057945)

[Supplementary Appendix 2 – Link to Protocol 9](#_Toc227057946)

[Supplementary Appendix 3 – Known Studies Prior to Screening 9](#_Toc227057947)

[Supplementary Appendix 4 – Studies Considered for Inclusion in the Updated Search 10](#_Toc227057948)

# Supplementary Appendix 1 Search Strategy

**Medline (1946 to 2025 Sep 11)**

1. factor Xa inhibitor.mp. or exp Factor Xa Inhibitors/
2. apixaban.mp.
3. Eliquis.mp.
4. betrixaban.mp.
5. edoxaban.mp.
6. rivaroxaban.mp.
7. darexaban.mp.
8. otamixaban.mp.
9. direct oral anticoagulant*.mp. or direct oral anticoag*.mp.
10. DOAC*.mp.
11. Novel oral anticoagulant*.mp.
12. New oral anticoagulant*.mp.
13. NOAC*.mp.
14. 1 or 2 or 3 or 4 or 5 or 6 or 7 or 8 or 9 or 10 or 11 or 12 or 13
15. exp Catheter Ablation/ or exp Radiofrequency Ablation/ or exp Ablation Techniques/ or exp High-Intensity Focused Ultrasound Ablation/ or ablation.mp.
16. exp Cardiac Pacing, Artificial/ or exp Pacemaker, Artificial/ or pacemaker.mp.
17. Coronary angiography.mp. or exp Coronary Angiography/
18. exp Dermatologic Surgical Procedures/ or dermatologic procedure*.mp.
19. exp Dental Care/ or dental procedure*.mp.
20. exp Defibrillators, Implantable/ or cardioverter-defibrillator.mp.
21. Phacoemulsification.mp. or exp Cataract Extraction/ or exp Phacoemulsification/
22. 15 or 16 or 17 or 18 or 19 or 20 or 21
23. exp randomized controlled trial/ or controlled clinical trial.pt. or randomized.ab. or placebo.ab. or drug therapy.fs. or randomly.ab. or trial.ab. or groups.ab.
24. exp animals/ not humans.sh.
25. 23 not 24
26. 14 and 22 and 25

**Embase (1974 to 2024 October 07)**

1. factor Xa inhibitor.mp. or exp blood clotting factor 10a inhibitor/
2. apixaban.mp. or exp apixaban/
3. eliquis.mp.
4. betrixaban.mp.
5. edoxaban.mp.
6. rivaroxaban.mp. or exp rivaroxaban/
7. darexaban.mp.
8. otamixaban.mp. or exp otamixaban/
9. direct oral anticoag*.mp.
10. DOAC*.mp.
11. Novel oral anticoag*.mp.
12. New oral anticoag*.mp.
13. NOAC*.mp.
14. 1 or 2 or 3 or 4 or 5 or 6 or 7 or 8 or 9 or 10 or 11 or 12 or 13
15. exp laser ablation system/ or exp thermal ablation/ or exp radiofrequency ablation device/ or exp ablation therapy/ or ablation.mp. or exp radiofrequency ablation/ or exp catheter ablation/ or exp ablation device/ or exp ablation catheter/ or exp radiofrequency catheter ablation/
16. pacemaker.mp. or exp cardiac rhythm management device/
17. exp coronary angiography/ or exp angiocardiography/ or coronary angiograph*.mp.
18. exp coronary angiography/ or exp angiocardiography/ or coronary angiograph*.mp.
19. exp dermatological procedure/ or dermatologic procedure*.mp. or exp skin surgery/
20. exp dental procedure/ or dental procedure*.mp.
21. exp defibrillator pacemaker/ or exp implantable cardioverter defibrillator/ or defibrillator.mp. or exp dual chamber implantable cardioverter defibrillator/ or exp defibrillator/ or exp biventricular implantable cardioverter defibrillator/
22. Phacoemulsification.mp. or exp cataract extraction/ or exp phacoemulsification/
23. 15 or 16 or 17 or 18 or 19 or 20 or 21 or 22
24. exp randomized controlled trial/ or controlled clinical trial/ or random$.ti,ab. or randomization/ or intermethod comparison/ or placebo.ti,ab. or (compare or compared or comparison).ti,ab.
25. ((evaluated or evaluate or evaluating or assessed or assess) and (compare or compared or comparing or comparison)).mp.
26. ((open adj label) or ((double or single or doubly or singly) adj (blind or blinded or blindly))).ti,ab. or double blind procedure/ or parallel group$1.ti,ab. or (crossover or cross over).ti,ab. or ((assign$ or match or matched or allocation) adj5 (alternate or group$1 or intervention$1 or patient$1 or subject$1 or participant$1)).ti,ab. or (assigned or allocated).ti,ab. or (controlled adj7 (study or design or trial)).ti,ab. or (volunteer or volunteers).ti,ab. or human experiment/ or trial.ti.
27. 24 or 25 or 26
28. (random$ adj sampl$ adj7 ("cross section$" or questionnaire$1 or survey$ or database$1)).ti,ab. not (comparative study/ or controlled study/ or randomi?ed controlled.ti,ab. or randomly assigned.ti,ab.)
29. cross-sectional study.mp. not (exp randomized controlled trial/ or controlled clinical trial/ or controlled study/ or randomi?ed controlled.ti,ab. or control group$1.ti,ab.)
30. ((case adj control$).mp. and random$.ti,ab.) not randomi?ed controlled.ti,ab.
31. (systematic review not (trial or study)).ti,ab.
32. (nonrandom$ not random$).ti,ab.
33. "random field$".ti,ab.
34. (random cluster adj3 sampl$).ti,ab.
35. (review.ab. and review.pt.) not trial.ti.
36. "we searched".ab. and (review.ti. or review.pt.)
37. "update review".ab.
38. (databases adj4 searched).ab.
39. (rat or rats or mouse or mice or swine or porcine or murine or sheep or lambs or pigs or piglets or rabbit or rabbits or cat or cats or dog or dogs or cattle or bovine or monkey or monkeys or trout or marmoset$1).ti. and animal experiment/
40. animal experiment/ not (human experiment/ or human/)
41. 28 or 29 or 30 or 31 or 32 or 33 or 34 or 35 or 36 or 37 or 38 or 39 or 40
42. 27 not 41
43. 14 and 23 and 42

**Central (1933 to 2025 Sep 11)**

ID Search Hits

1. (DOAC):ti,ab,kw (Word variations have been searched) 570
2. (direct oral anticoagulant):ti,ab,kw (Word variations have been searched) 1349
3. (apixaban):ti,ab,kw (Word variations have been searched) 1325
4. ("rivaroxaban"):ti,ab,kw (Word variations have been searched) 2445
5. (direct oral anticoag*):ti,ab,kw (Word variations have been searched) 1353
6. (NOAC):ti,ab,kw (Word variations have been searched) 464
7. (novel oral anticoag*):ti,ab,kw (Word variations have been searched) 370
8. (new oral anticoag*):ti,ab,kw (Word variations have been searched) 731
9. #1 or #2 or #3 or #4 or #5 or #6 or #7 or #8 4838
10. (ablation):ti,ab,kw (Word variations have been searched) 13191
11. ("pacemaker"):ti,ab,kw (Word variations have been searched) 3635
12. ("cardiac pacing"):ti,ab,kw (Word variations have been searched) 1494
13. ("coronary angiography"):ti,ab,kw (Word variations have been searched) 9675
14. ("dermatologic procedure"):ti,ab,kw (Word variations have been searched) 158
15. ("dermatological procedure"):ti,ab,kw (Word variations have been searched) 158
16. (dental procedure):ti,ab,kw (Word variations have been searched) 10194
17. ("defibrillator"):ti,ab,kw (Word variations have been searched) 4929
18. ("phacoemulsification"):ti,ab,kw (Word variations have been searched) 3824
19. #10 or #11 or #12 or #13 or #14 or #15 or #16 or #17 or #18 44900
20. #9 and #19 301

**CINAHL (1951 to 2025 Sep 11)**

1. S1 direct oral anticoagulant OR direct oral anticoag* 2,030
2. S2 DOAC OR apixaban OR rivaroxaban 4,484
3. S3 NOAC OR new oral anticoagulant OR novel oral anticoagulant 1,597
4. S4 S1 OR S2 OR S3 Expanders - Apply equivalent subjects 6,438
5. S5 ablation OR ( pacemaker or artificial cardiac pacemaker ) OR cardiac pacing 54,524
6. S6 coronary angiography 21,326
7. S7 dermatologic procedure 71
8. S8 dermatology procedure 10
9. S9 dermatologic surgery 165
10. S10 dental procedure 1,328
11. S11 defibrillator OR phacoemulsification 20,839
12. S12 S5 or S6 or S7 or S8 or S9 or S10 or S11 93,064
13. S13 (S5 or S6 or S7 or S8 or S9 or S10 or S11) AND (S4 AND S12) 30

# Supplementary Table 1 – Summary Table of Subgroup Analyses

| Outcome | Type of Procedure | | | Study Design | | | | Interruption Interval | | | | |
| --- | --- | --- | --- | --- | --- | --- | --- | --- | --- | --- | --- | --- |
|  | Ablation Studies (Patients) | Device Insertion Studies (Patients) | Test of Interaction – P Value | | Nonrandomized Studies (Patients) | Randomized Studies (Patients) | Test of Interaction – P Value | | Day of Studies (Patients) | 24-Hour Studies (Patients) | 48-Hour Studies (Patients) | Test of Interaction – P Value |
| Thrombotic Events | 13 studies  (n = 4739) | 4 studies  (n = 2149) | 0.78 | | 7 studies  (n = 2855) | 8 studies  (n = 2926) | 0.82 | | 6 studies  (n = 2196) | 8 studies  (n = 2174) | 3 studies  (n = 3278) | 0.96 |
| Total Bleeding Events | 11 studies  (n = 3844) | 4 studies  (n = 1397) | 0.63 | | 12 studies  (n =3112) | 8 studies  (n = 3109) | **0.03*** | | 6 studies  (n = 2258) | 8 studies  (n = 1984) | 4 studies  (n = 1422) | 0.58 |
| Minor Bleeding Events | 8 studies  (n = 2611) | 1 study  (n = 662) | 0.77 | | 6 studies  (n = 1503) | 6 studies  (n = 2684) | 0.37 | | 4 studies  (n = 1923) | 6 studies  (n = 1557) | 1 study  (n = 662) | 0.52 |
| Major Bleeding Events | 10 studies  (n = 3270) | 4 studies  (n = 1397) | 0.75 | | 11 studies  (n = 3063) | 8 studies  (n = 3109) | 0.16 | | 5 studies  (n = 2256) | 7 studies  (n = 1935) | 3 studies  (n = 1302) | 0.93 |
| Adverse Events | 5 studies  (n = 1132) | 3 studies  (n = 823) | 0.21 | | 4 studies  (n = 635) | 5 studies  (n = 1386) | 0.88 | | 1 study  (n = 200) | 5 studies  (n = 1033) | 2 studies  (n = 728) | 0.82 |
| Mortality | 1 study  (n = 295) | 1 study  (n = 101) | ID | | 2 studies  (n = 1299) | 2 studies  (n = 396) | ID | | 1 study  (n = 295) | 2 studies  (n = 921) | 0 studies  (n = 0) | ID |

* - indicates statistical significance; ID – insufficient data

# Supplementary Table 2 – ICEMAN Table for Credibility of Subgroup Analyses

| Criteria | Subgroup Effects | | | | | | | | |
| --- | --- | --- | --- | --- | --- | --- | --- | --- | --- |
|  | Thrombotic Events and Procedure Type | Thrombotic Events and Study Design | Thrombotic Events and DOAC Timing | All Bleeding and Procedure Type | All Bleeding and Study Design | All Bleeding and DOAC Timing | Minor Bleeding and Procedure Type | Minor Bleeding and Study Design | Minor Bleeding and DOAC Timing |
| 1: Is the analysis of effect modification based on comparison within rather than between trials? | Between Trials | Between Trials | Between Trials | Between Trials | Between Trials | Between Trials | Between Trials | Between Trials | Between Trials |
| 2: For within-trial comparisons, is the effect modification similar from trial to trial? | NA | NA | NA | NA | NA | NA | NA | NA | NA |
| 3: For between-trial comparisons, is the number of trials large? | Rather small  (4 studies) | Rather large  (8 studies) | Rather small  (3 studies) | Rather small  (4 studies) | Rather large  (8 studies) | Rather small  (4 studies) | Very small (1 study) | Very small (2 studies) | Very small (1 study) |
| 4: Was the direction of effect modification correctly hypothesized a priori? | NA | NA | NA | NA | NA | NA | NA | NA | NA |
| 5: Does a test for interaction suggest that chance is an unlikely explanation of the apparent effect modification? | Chance very likely | Chance very likely | Chance very likely | Chance very likely | **Chance likely**  **(p = 0.02)** | Chance very likely | Chance very likely | Chance very likely | Chance very likely |
| 6: Did the authors test only a small number of effect modifiers or consider the number in their  statistical analysis? | Probably yes  (3 factors) | Probably yes  (3 factors) | Probably yes  (3 factors) | Probably yes  (3 factors) | Probably yes  (3 factors) | Probably yes  (3 factors) | Probably yes  (3 factors) | Probably yes  (3 factors) | Probably yes  (3 factors) |
| 7: Did the authors use a random effects model? | Definitely Yes | Definitely Yes | Definitely Yes | Definitely Yes | Definitely Yes | Definitely Yes | Definitely Yes | Definitely Yes | Definitely Yes |
| 8: If the effect modifier is a continuous variable, were arbitrary cut points avoided? | NA | NA | NA | NA | NA | NA | NA | NA | NA |
| **Overall Credibility** | **Low** | **Low** | **Low** | **Low** | **Moderate** | **Low** | **Very Low** | **Very Low** | **Very Low** |

# Supplementary Table 3 – ICEMAN Table for Credibility of Subgroup Analyses

| Criteria | Subgroup Effects | | | | | | | | |
| --- | --- | --- | --- | --- | --- | --- | --- | --- | --- |
|  | Major Bleeding and Procedure Type | Major Bleeding and Study Design | Major Bleeding and DOAC Timing | Adverse Events and Procedure Type | Adverse Events and Study Design | Adverse Events and DOAC Timing | Mortality and Procedure Type | Mortality and Study Design | Mortality and DOAC Timing |
| 1: Is the analysis of effect modification based on comparison within rather than between trials? | Between Trials | Between Trials | Between Trials | Between Trials | Between Trials | Between Trials | Between Trials | Between Trials | Between Trials |
| 2: For within-trial comparisons, is the effect modification similar from trial to trial? | NA | NA | NA | NA | NA | NA | NA | NA | NA |
| 3: For between-trial comparisons, is the number of trials large? | Rather small  (4 studies) | Rather large  (8 studies) | Rather small  (3 studies) | Rather small  (3 studies) | Rather small  (4 studies) | Very Small  (1 study) | Very Small  (2 studies) | Rather Small  (4 studies) | Rather Small  (3 studies) |
| 4: Was the direction of effect modification correctly hypothesized a priori? | NA | NA | NA | NA | NA | NA | NA | NA | NA |
| 5: Does a test for interaction suggest that chance is an unlikely explanation of the apparent effect modification? | Chance very likely | Chance very likely | Chance very likely | Chance very likely | Chance very likely | Chance very likely | Chance very likely | Chance very likely | Chance very likely |
| 6: Did the authors test only a small number of effect modifiers or consider the number in their  statistical analysis? | Probably yes  (3 factors) | Probably yes  (3 factors) | Probably yes  (3 factors) | Probably yes  (3 factors) | Probably yes  (3 factors) | Probably yes  (3 factors) | Probably yes  (3 factors) | Probably yes  (3 factors) | Probably yes  (3 factors) |
| 7: Did the authors use a random effects model? | Definitely Yes | Definitely Yes | Definitely Yes | Definitely Yes | Definitely Yes | Definitely Yes | Definitely Yes | Definitely Yes | Definitely Yes |
| 8: If the effect modifier is a continuous variable, were arbitrary cut points avoided? | NA | NA | NA | NA | NA | NA | NA | NA | NA |
| **Overall Credibility** | **Low** | **Low** | **Low** | **Low** | **Low** | **Very Low** | **Very Low** | **Low** | **Low** |

# Supplementary Appendix 2 – Link to Protocol

PROSPERO Link: <https://www.crd.york.ac.uk/PROSPERO/view/CRD42024621729>

# Supplementary Appendix 3 – Known Studies Prior to Screening

1. Nakamura K, Naito S, Sasaki T, et al. Uninterrupted vs. interrupted periprocedural direct oral anticoagulants for catheter ablation of atrial fibrillation: a prospective randomized single-centre study on post-ablation thrombo-embolic and haemorrhagic events. *Europace : European pacing, arrhythmias, and cardiac electrophysiology : journal of the working groups on cardiac pacing, arrhythmias, and cardiac cellular electrophysiology of the European Society of Cardiology*. 2019;21(2):259-267. doi:[10.1093/europace/euy148](https://doi.org/10.1093/europace/euy148).
2. Yamaji H, Murakami T, Hina K, et al. Activated clotting time on the day of atrial fibrillation ablation for minimally interrupted and uninterrupted direct oral anticoagulation therapy: Sequential changes, differences among direct oral anticoagulants, and ablation safety outcomes. *Journal of cardiovascular electrophysiology*. 2019;30(12):2823-2833. doi:[10.1111/jce.14260](https://doi.org/10.1111/jce.14260).
3. Sheikh MA, Kong X, Haymart B, et al. Comparison of temporary interruption with continuation of direct oral anticoagulants for low bleeding risk procedures. *Thromb Res*. 2021;203:27-32. doi:[10.1016/j.thromres.2021.04.006](https://doi.org/10.1016/j.thromres.2021.04.006).
4. Nagao T, Suzuki H, Matsunaga S, et al. Impact of periprocedural anticoagulation therapy on the incidence of silent stroke after atrial fibrillation ablation in patients receiving direct oral anticoagulants: uninterrupted vs. interrupted by one dose strategy. *Europace : European pacing, arrhythmias, and cardiac electrophysiology : journal of the working groups on cardiac pacing, arrhythmias, and cardiac cellular electrophysiology of the European Society of Cardiology*. 2019;21(4):590-597. doi:[10.1093/europace/euy224](https://doi.org/10.1093/europace/euy224).
5. Izzetti R, Cinquini C, Nisi M, Mattiozzi M, Marotta M, Barone A. Direct Oral Anticoagulants and Bleeding Management Following Tooth Extractions—A Prospective Cohort Study. *Dent J (Basel)*. 2024;12(9):279. doi:[10.3390/dj12090279](https://doi.org/10.3390/dj12090279).

# Supplementary Appendix 4 – Studies Considered for Inclusion in the Updated Search

1. Johansson K, Becktor JP, Naimi-Akbar A, Svensson PJ, Götrick B. Continuous use of direct oral anticoagulants during and after simple and surgical tooth extractions: a prospective clinical cohort study. *BMC Oral Health*. 2025;25(1):554. doi:[10.1186/s12903-025-05949-9](https://doi.org/10.1186/s12903-025-05949-9).
   1. Excluded due to inclusion of only patients on continuous DOACs, not interrupted.
2. Mahé I, Hajage D, Monnet–Corti V, et al. Oral anticoagulant periprocedural management in patients undergoing an oral, dental implant or periodontal surgery: a prospective national observational survey. *Research and Practice in Thrombosis and Haemostasis*. 2025;9(3):102848. doi:[10.1016/j.rpth.2025.102848](https://doi.org/10.1016/j.rpth.2025.102848).
   1. Excluded due to inclusion of patients undergoing complex dental procedures (bone grafting, sinus lift, mucosal/bone surgery, or other procedures at bleeding risk.
3. Sanz-Sánchez J, Chiarito M, Calderón AT, et al. Uninterrupted Direct-Acting Oral Anticoagulation in Patients Undergoing Transradial Percutaneous Coronary Procedures: The DOAC-NOSTOP. *Catheterization and Cardiovascular Interventions*. 2025;106(2):1371-1377. doi:[10.1002/ccd.31691](https://doi.org/10.1002/ccd.31691)
   1. Excluded due to inclusion of patients receiving coronary angiography via femoral access, not radial as described by *Douketis and Spyropoulos, 2024*.
4. Lee SR, Lee KY, Park JS, et al. Perioperative Factor Xa Inhibitor Discontinuation for Patients Undergoing Procedures With Minimal or Low Bleeding Risk. JAMA Netw Open. 2025;8(2):e2458742. doi:10.1001/jamanetworkopen.2024.58742
   1. Excluded due to the lack of a continuous comparison arm.
